# Decision-Making Under Risk and Uncertainty by Substance Abusers and Healthy Controls

**DOI:** 10.3389/fpsyt.2021.788280

**Published:** 2022-01-28

**Authors:** Diana Mejía, Laurent Avila-Chauvet, Aldebarán Toledo-Fernández

**Affiliations:** ^1^Psychology Department, Instituto Tecnológico de Sonora (ITSON), Ciudad Obregón, Mexico; ^2^Facultad de Psicología, Universidad Anáhuac México, Ciudad de México, Mexico

**Keywords:** decision making, discounting, risk-taking, substance abuse, methamphetamine

## Abstract

Cognitive impairment characterized by high impulsivity and risk-taking has been correlated with substance-related disorders. However, it is unclear if the decision-making process is well known upon consideration of factors such as uncertainty environments, risk, and time manipulation in different decision-making procedures. The main objective of this study was to identify behavioral differences between substance abusers and healthy control participants in a behavioral test battery, including (1) two uncertainty decision-making tasks, the Balloon Analog Risk Task (BART) and the Iowa Gambling Task (IGT, trial 1–40); (2) three risk-taking tasks, the Columbia Card Task Hot version (CCT-hot), Columbia Card Task Cold version (CCT-cold), and the IGT (trial 41–100); and (3) an impulsivity task, the Delay Discounting task (DD). The second objective looked at how the six behavioral tests correlate. We worked with a sample of 54 adult participants (*Substance abusers*: *n* = 28; *Healthy controls*: *n* = 26). An anonymous survey website was created to execute all the cognitive tasks. The results showed no statistically significant differences between the groups in any of the tasks. However, the results showed an upward trend of impulsive (i.e., steeply discounting curve) and risk-taking behaviors (i.e., a low learning curve in IGT) in substance abuse participants. The factor analysis results showed four different main factors: (1) risk-taking task in the IGT (trial 40–100), (2) uncertainty task in BART, (3) impulsivity in DD, IGT (trial 1–40), and (4) deliberate process in the Columbia card task (cold and hot). We conclude that factors such as the uncertainty tasks in the BART and the first block of IGT trials, the risk cues in the CCT tasks (i.e., number of loss, number of gains, and loss cards), and the time to delivery in the DD task, can affect the complex decision-making process in both clinical and healthy groups.

## Introduction

Several studies have found mental health complications, including cognitive functioning by substance use disorders (SUDs). For example, one of the most affected cognitive abilities is decision-making (1, 2). Recent studies have demonstrated that substance abusers who are in treatment (alcohol, cocaine, heroin, tobacco, and methamphetamine) exhibit extreme values of choosing small immediate rewards over large delay rewards (i.e., steeply delay discounting) (3–5), and risk-taking behaviors (6–8). Nevertheless, it remains unclear whether risk-taking behaviors are associated with impulsivity (i.e., steeply delay discounting).

For instance, some studies have found no differences between control and substance abuse groups when using a risk measure such as probability discounting (choosing larger, riskier reward over a smaller, less risky reward) (9–12). Nevertheless, risky behavior using the Iowa Gambling Task (IGT, task of four decks with different probabilities of losses), the results displayed lower chose of advantage cards of money across substance abusers than participants in the control conditions (2, 5). However, in the IGT task, (13) found that meth dependent individuals who were abstinent for longer periods exhibited better choice of advantage cards than those who were abstinent for shorter periods. Therefore, abstinence time seems like an important variable to consider besides the kind of task to measure risk-taking to contrast substance abusers and healthy controls.

Other studies have shown statistically significant differences while employing risk-taking tasks such as the Columbia Card Task Hot version (CCT-hot) and the Columbia Card Task Cold version (CCT-cold). The results of these tasks demonstrated significant risky behaviors (selecting more cards in a risk environment) associated with substance abusers in comparison to the control conditions in both tests (14, 15). Another important task to measure risk-taking is the Balloon Analog Risk Task (BART) (select pumps with different probabilities). This task has similarly reported that substance abusers are prone to select more pumps in contrast to healthy control participants (6, 8). However, upon evaluating risk-taking behavior and impulsivity in substance abusers, the correlation among several behavioral tasks remains unclear. For example, related studies have reported no correlation (16) or only moderate correlations (17) among the delay discounting task (DD), IGT, and BART tests.

Furthermore, few studies have explored differences between risk-taking tasks and uncertainty decision-making tasks within the same population sample. For example, De Groot and Thurik (18) argued that the BART test should not be considered a behavioral measure of risk-taking. This was argued because awareness regarding environmental risk cues are not made explicit in participants during the trials. Some studies have argued that instead of measuring risk-taking, the IGT procedure measures uncertainty decision-making task during the 1 to 40 trials. In addition, it has been argued that the IGT procedure only measures risk-associated behaviors when participants have access to information regarding the four-card decks (trials 41–100) (19, 20).

Concerning CCT-hot and CCT-cold, the participants are given several pieces of information during the beginning of the task. This information influences participants' decision on how many cards to turn over. Such feedback processing factors and environmental contingencies are part of risk-taking measures (21). Buelow and Blaine (20) calculated the correlation values among the IGT, BART, CCT-hot, and CCT-cold procedures. During these procedures, the team found four separate processes with a factor analysis (BART, CCT, IGT-I, IGT-II), and with either no correlation or low correlations among the tasks, just like other studies [(22, 23)].

Interestingly, the IGT demonstrated two different factors: the first factor was for trials 1 to 40 (uncertainty task), and the second factor was for trials 41–100 (risk-taking task). In this same task, Jollant et al. (24) reported increased significant differences between healthy controls and clinical patients during trials 41–100. These findings in the clinical settings are significant as they suggest that the performance of several tasks should be contrasted to identify cognitive impairments. It is unclear whether the results are consistent among substance abusers who are faced with risk-taking and uncertainty tasks.

Therefore, the main objective of this study was to identify behavioral differences between substance abusers and healthy control participants during a behavioral test battery and performance during the task's conditions (IGT, BART, CCT-cold and CCT-hot). The behavioral test battery consisted of: (1) two uncertainty tasks (i.e., BART, IGT trial 1–40); (2) three risk-taking tasks (i.e., CCT-hot, CCT-cold, IGT trial 41–100); (3) and an impulsivity task (i.e., DD task). The second objective of this study was to analyze the correlation between the behavioral tests, and to identify predictive factors of substance abuse. Only a few studies have included all these elements within the same study. Additionally, as a third objective, we performed a Classification and Regression Trees (CART) algorithm to identify predictive factors of substance abuse in each behavioral task.

As in the studies mentioned, we expected (1) that participants in the clinical sample will show more risk-taking and impulsivity behavior in comparison to participants in the healthy controls; (2) a positive correlation between the uncertainty tasks (BART and IGT 1–40 trials; (3) a positive correlation among the risk-taking tasks (IGT 41–100 trials, CCT-hot, CCT-cold); and finally, (4) a positive correlation among uncertainty tasks, risk-taking tasks, and impulsivity tasks (DD task).

## Method

### Participants

We worked with 54 male adult participants, from which 28 participants were substance abusers enrolled in the treatment center, (*age*: *M* = 32.07, *SD* = 14.10), and 26 participants as healthy controls (*age*: *M* = 23.08, *SD* = 12.61).

All of the substance abuse participants were in a residential addiction treatment center in the north of Sinaloa (Mexico) and were actively involved in treatment. The participants were in the second and third week of the treatment, and they did not have a report in their files of withdrawal symptoms in the 72 previous hours. The treatment consisted in 3 months of cognitive behavioral training and motivational interviewing strategies [see Barragán et al. (25) for details].

Participants from the control condition were community college students and/or friends or relatives of psychology students. The participants in the control group did not have any psychiatric diagnoses, neurological diagnoses, and substance use disorder. All the students enrolled in the psychology course received extra credit for their participation. All participants were living in the city at the time of the study.

The Sonora Institute of Technology Institutional Review Board (ID 84) approved the protocol. Additionally, all participants provided written informed consent following the Declaration of Helsinki. Participants were not compensated with money for their participation. The informed consent declared that participants would remain blinded to the study hypotheses and groupings.

### Measures

A set of self-report structured surveys was used to collect information regarding demographics (i.e., gender, region, age, education in years, income, psychiatric diagnoses, neurological diagnoses), drug usage, type of drugs used, frequency of tobacco and marijuana (cigarette) consumption, frequency of alcoholic drinks consumption, the number of drugs used, and frequency of usage during the week. Information concerning safety conduct related to COVID-19, such as preventive and adherence behaviors based on the WHO (26) safety guidelines and recommendations were also collected from participants. As COVID-19 safety recommendations and enforced restrictions overlapped with data collection, and to avoid participant withdrawal from the study, it was decided that the online survey should remain short in length (between 15 and 30 min).

### Behavioral Tasks

All behavioral tasks were programmed using the cross-platform development environment GameMaker Studio (v. 1.4).

#### Delay Discounting Task (DD)

The DD procedure consisted of one block of four training trials and one block of thirty-five testing trials. For each delay, there were seven trials. The delays included a week, a month, 6 months, a year, and 3 years. The initial value of the large alternative was two hundred Mexican pesos. The discounting task used an adjusting-amount system that converges on the amount of an immediate—certain outcome equal in subjective value—to a delayed or probabilistic outcome [for a detailed description, see Du et al. (27)]. The dependent variable used to measure how participants discounted the value of the delay was the Area Under the Discounting Curve (AUC). The AUC was calculated directly from the empirical discounting curve (i.e., the observed indifference points). These values provide an atheoretical measure of how 'steeply' participants discount a specific outcome. In which case, 0.0 indicated the maximum theoretical discount value, and 1.0 indicated the minimum discount value [for a detailed description, see Myerson et al. (28)]. Steep discounting of delayed rewards is frequently equated with impulsivity or a lack of self-control (29).

#### Balloon Analog Risk Task (BART)

BART is a computerized task designed for users to accumulate a greater number of points without exploding the balloon. Participants must decide to secure their winnings or otherwise risk losing their accumulated earnings. Each time participants pressed on the balloon's image, they received five points. With each balloon press, the probability of the balloon exploding increased, as did the likelihood of losing accumulated points. The dependent variable was the average frequency of pumps adjusted to the unexploded balloons by probabilities type *I* (1/8), *II* (1/32), *III* (1/128) (30).

#### Cold and Hot Columbia Card Task (CCT-Cold, CCT-Hot)

The CCT is a computerized measure of risk-taking behavior. The goal of the CCT task is to earn a greater number of points by flipping cards (total = 32) in a virtual deck. In the CCT-cold version, participants received trial-by-trial information about the number of “loss” cards (either 1 or 3), the number of points that could be won on each card (10 or 30 points), and the number of points that could be lost if a “loss” card was chosen (250 or 750 points). Then, participants indicated the total number of cards to be turned over. Participants did not receive feedback on their selections until the end of the task, which totaled 24 trials. The information obtained in the CCT-hot version was comparable to the information obtained in the CCT-cold version. However, in the hot version, participants manually clicked on each card to flip it over. Manual clicking provided immediate feedback regarding the type of outcome for each card, either winning or losing. This action also revealed the number of points earned (or lost) during each card selection, and the total points lost if a loss card was later selected. For both the CCT-hot and the CCT-cold, the average number of cards chosen was used as an outcome variable, with higher scores indicating riskier performances (22).

#### Iowa Gambling Task (IGT)

The goal of this computerized task is to evaluate the decision-making process in situations of uncertainty and risk. The IGT consisted of four virtual card decks that were displayed to each participant. Participants could choose one card from one of the four decks. The decks differed in rewards magnitude, punishments magnitude, and probability of punishments. Decks of lesser magnitude and penalties were more advantageous in the long term than decks of greater magnitude and penalties. Participants were instructed to maximize their profits by selecting 100 cards from one of the four decks (*A, B, C, D*). Decks *A* and *B* were disadvantageous, while decks *C* and *D* were advantageous (31). For the present study, the task was divided into five blocks of 20 trials. The proportion of advantageous choices was calculated for each block, where one means that participants only chose the advantageous alternatives, while zero means that participant only chose the disadvantageous alternatives. We contrasted the average proportion of advantageous choices by groups wherein the lesser proportion of total scores indicated riskier performances.

### General Procedures

An anonymous survey website (https://lcaa.com.mx/Exp3/) was created for this study. Data collection (i.e., behavioral battery tests and demographic information) was carried out between August 2021 and September 2021. All participants used the same website. However, study procedures differed between groups concerning how participants accessed the behavioral tests.

For healthy control participants, the website link was sent *via* email. The informed consent, including the study's purpose, was presented to participants before data collection.

Due to COVID-19 restrictions, assessments with clinical participants were restricted to a period of 60 min. Substance abuse participants accessed the website through a tablet, which was provided by researchers at the drug rehab center. Participants in the substance abuse group were referred to this study by the center's head psychiatrist. Assessments took place in the psychology offices of the drug rehab facilities. To assess participants in the substance abuse group, participants were required to meet the inclusion criteria. These criteria included having no withdrawal symptoms, schizophrenia events, or other psychotic disorders reported by the psychiatrist in their file. Once selected to participate, the researcher read the informed consent aloud to the participant and then assigned an ID folio.

After agreeing to participate in the study, the participants started the behavioral test battery in the following order: BART, DD, IGT, CCT-hot, and CCT-cold. The time spent during the evaluation was approximately 45 minutes per participant.

### Data Analysis

This study used a cross-sectional design. Frequencies, means, and standard deviations were calculated for all sample demographic characteristics.

To determine whether a parametric or nonparametric statistical test for correlations and intergroup comparison would be used, researchers carried out a normality analysis including a Kolmogorov-Smirnov test and Shapiro-Wilk test. In addition, Levene's test was used to evaluate the homogeneity of variances for each dependent variable. The normality results suggested using a nonparametric test.

Concerning the first aim of this study, we first identify differences concerning demographic variables and decision-making tasks by group. We used the Mann–Whitney U test to contrast the performance of substance abusers and healthy control groups for the dependent variables in each of the behavioral tasks (DD, BART, CCT-hot, CCT-cold, and IGT). The test also evaluated differences between age, education, income levels, frequency of tobacco and marijuana (cigarette) use, frequency of alcoholic beverage consumption, and the quantity of methamphetamine used (grams).

In order to understand each task individually, a Friedman test for repeated measures was used to compare the performance inside each task: BART, IGT, CCT-hot, and CCT-cold tasks. In this regard, for the IGT, the dependent variable was the average of advantageous selection by each block of 20 trials, and the independent variable was the five blocks. For the BART, the dependent variable was the average number of adjusted balloons and the independent variable was the three probabilities. For the CCT-hot and CCT-cold, the dependent variable was average number of card selections and the independent variable was loss cards, lose points and points gained.

To fit the hyperboloid function of the delay discounting task, we used the equation: *V*=1/(1+*bX*), where *V* is the subjective value of the delayed outcome, *b* is a parameter reflecting the discount rate at which the subjective value decreases as the delay until receiving the outcome increases, and *X* is the delay ((32)). A power function (*Y* = *ax*^*b*^) was used to analyze the participant's performance in the IGT test. Higher *b* values represented an increase in the preference for advantageous alternatives across the blocks (33).

About the second aim of this study, we initially performed a Pearson product-moment correlation and next a factor analysis to understand how decision-making variables might relate to each other. Principal components EFA with varimax rotation were used, with eigenvalues ≥1.00 retained. We followed the recommendation of MacCallum et al. (34) for the sample size. For this analysis, we used all samples.

Finally, to evaluate the degree of prediction of the decision-making tests and the belonging of the groups both logistic regression and Classification and Regression Trees (CART) algorithm were performed. Given the size of the sample, it was decided to build a decision tree with a maximum of 3 splits without cross validation. Each node describes decision rules, the number of cases, the probability of cases, and the Gini index. The lower Gini indexes suggest higher information gain or uncertainty reduction. The Gini index varies between values 0 and 1, where 0 expresses the purity of classification, and 1 indicates the random distribution of elements across various classes. The value of 0.5 of the Gini Index shows an equal distribution of elements over some classes. The predicted group is found in the leaves of each last node.

The statistical analyses were performed using SPSS v. 23® and MATLAB v. R2018b®.

## Results

### Group Characteristics

We worked with 54 adult participants. Their ages ranged between 18 and 51 years old (*M* = 27.74, *SD* = 14.03). The level of education was, on average, 12.30 years (*SD* = 6.25). When evaluating the most common type of drug used by the substance abuse group, the results showed two subgroups, polysubstance abusers (70%) and mono-drug abusers (30%). Crystal meth was the principal drug used by polysubstance abusers (81%). In comparison, the percentage of usage in mono-drug abusers was relatively smaller (44.4%). The abstinence time was on average 68.68 days (*SD* = 63.58). The analysis did not show significant differences between healthy controls and substance abusers in age (*p* = 0.058), income level (*p* = 0.397) and frequency of alcoholic drinks (*p* = 0.921). In contrast, there were statistically significant differences between the groups for the variables: level of education (*p* = 0*.0*01), frequency of tobacco (*p* < 0.001), marijuana (cigarettes) consumption (*p* < 0.001), and quantity of crystal meth consumption in grams (*p* < 0.001) (see Table 1).

**Table 1:** Group characteristics.

	**Healthy Controls**		**Substance abusers**		***Z***	***p***
***N***	**26**		**28**			
	***M***	***SD***	***M***	***SD***		
Age	23.08	12.61	32.07	14.10	−1.89	0.058
Level of education (years)	14.35	7.42	10.39	4.23	−3.19	0.001
Monthly income	$9,297.50	$10,478.55	$8,550.29	$14,308.34	−0.84	0.397
Number of tobacco (cigarettes)	1.81	5.29	12.3	11.4	−4.44	0.000
Number of marijuana (cigarettes)	0.44	1.96	2.22	2.43	−3.89	0.000
Number of alcohol drinks	4.92	6.39	5.07	6.66	−0.09	0.921
Quantity of crystal meth (grams)	0.12	0.32	2.21	1.89	−6.13	0.000

*A Mann–Whitney U test was implemented to contrast the variables between the groups*.

### Delay Discounting Results

**Figure 1:**
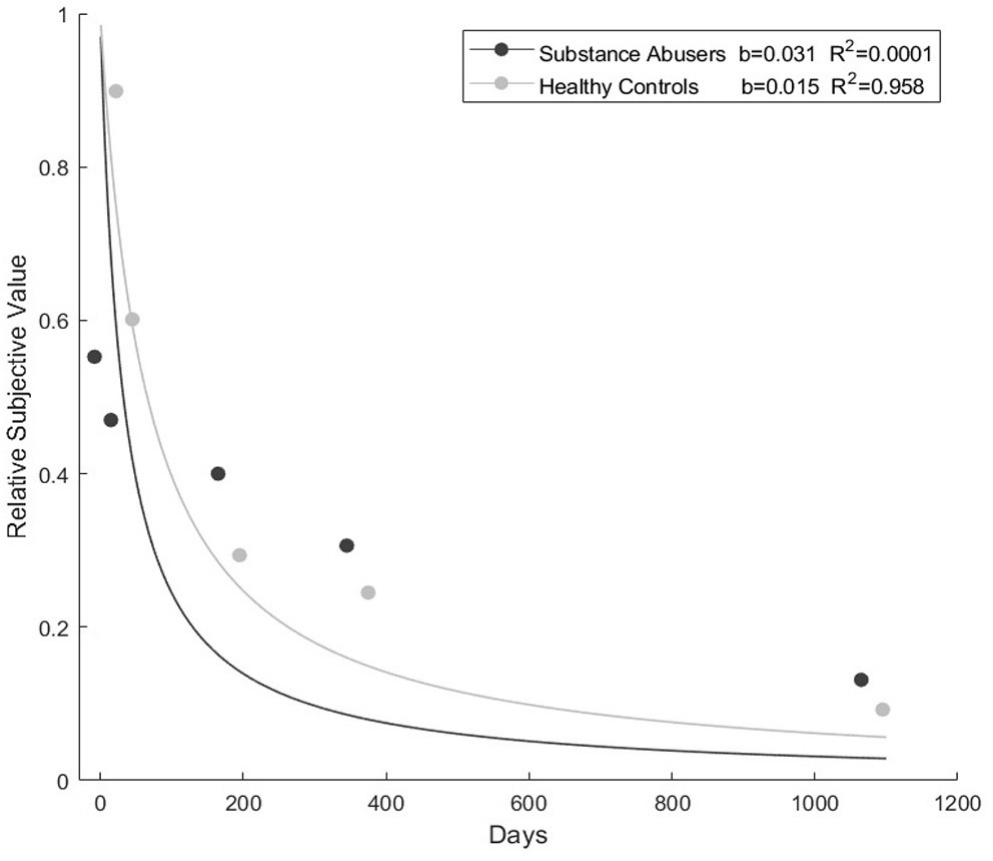
Discounting curve and fit to hyperboloid function.

The analysis also showed no differences between healthy controls and substance abusers in decision-making tasks (all *p*-values >0.05) (see Table 2). The fit of the hyperboloid function was adequate for the healthy controls (*R*^2^ = 0.958) in the delay discounting task. The *k* value was lower in healthy controls (*b* = 0.015) than substance abusers (*b* = 0.031) (see Figure 1). The substance abusers' group did not fit the hyperboloid equation.

**Table 2:** Means and standard deviations of the decision-making tasks.

		**DD**	**BART**			**IGT**					**CCT-hot**	**CCT-cold**
			**Type I**	**Type II**	**Type III**	**Block I**	**Block II**	**Block III**	**Block IV**	**Block V**		
Substance abusers	*M*	0.376	2.98	4.65	6.64	0.357	0.457	0.469	0.423	0.448	14.5	13.7
	*SD*	0.286	1.04	3.27	5.63	0.147	0.200	0.221	0.242	0.271	6.96	7.44
Healthy controls	*M*	0.278	2.95	5.30	6.95	0.319	0.394	0.415	0.423	0.446	14.3	11.3
	*SD*	0.214	1.33	3.44	4.85	0.219	0.244	0.339	0.312	0.328	5.62	8.49
	*Z*	−1.03	−0.139	−1.13	−0.675	−1.08	−1.20	−1.20	−0.095	−0.765	−0.208	−1.17
	*p*	0.299	0.890	0.257	0.500	0.277	0.229	0.228	0.924	0.444	0.835	0.264
	η^2^	0.020	0.000	0.024	0.008	0.022	0.027	0.027	0.000	0.011	0.000	0.025
	1-B	0.284	0.051	0.107	0.055	0.114	0.174	0.106	0.050	0.050	0.051	0.198

*IGT, Iowa Gambling Task, average of advantageous selection by 20-trials blocks; BART, Balloon Analog Risk Task, average number of adjusted balloons by probabilities type; CCT-cold and CCT-hot, Columbia Card Task, average number of card selections; DD, AUC of delay discounting*.

### Balloon Analog Risk Task Results

**Figure 2:**
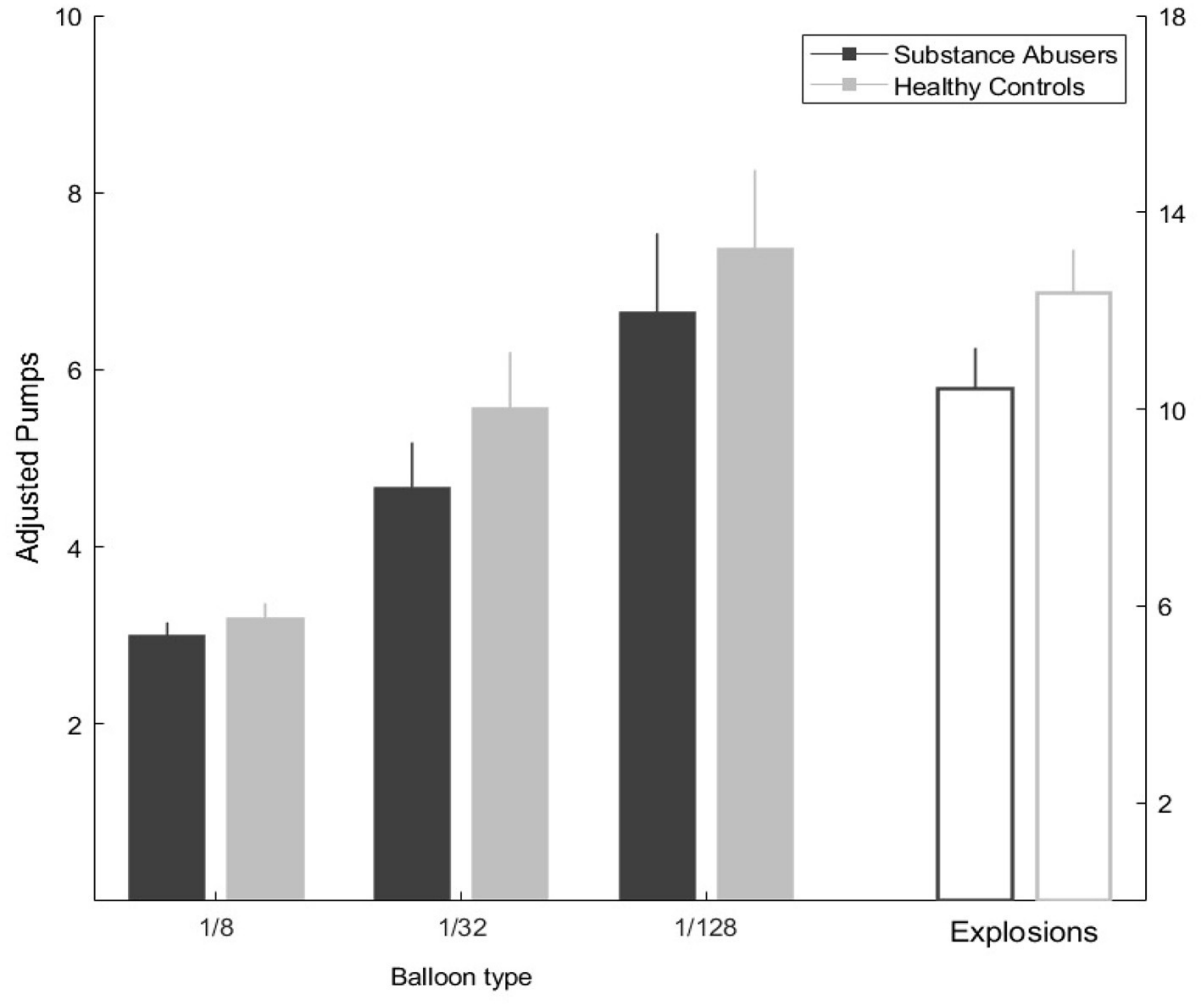
Balloon analog risk task performance in the three probabilities by group, and the total of balloon explosions during the task.

The analysis of the BART task demonstrated that the average performance among groups showed no statistically significant differences (*Z* = −1.22, *p* = 0.219). A repeated measures test was used to contrast the balloon probabilities of explosion used in the task demonstrated statistically significant differences in the task performance (substance abusers: χ^2^ = 8.85, *gl* = 2, *p* = 0.012, η^2^ = 0.327; healthy group: χ^2^ = 34.62, *gl* = 2, *p* = 0.000, η^2^ = 1.38) (see Figure 2).

### Iowa Gambling Task Results

**Figure 3:**
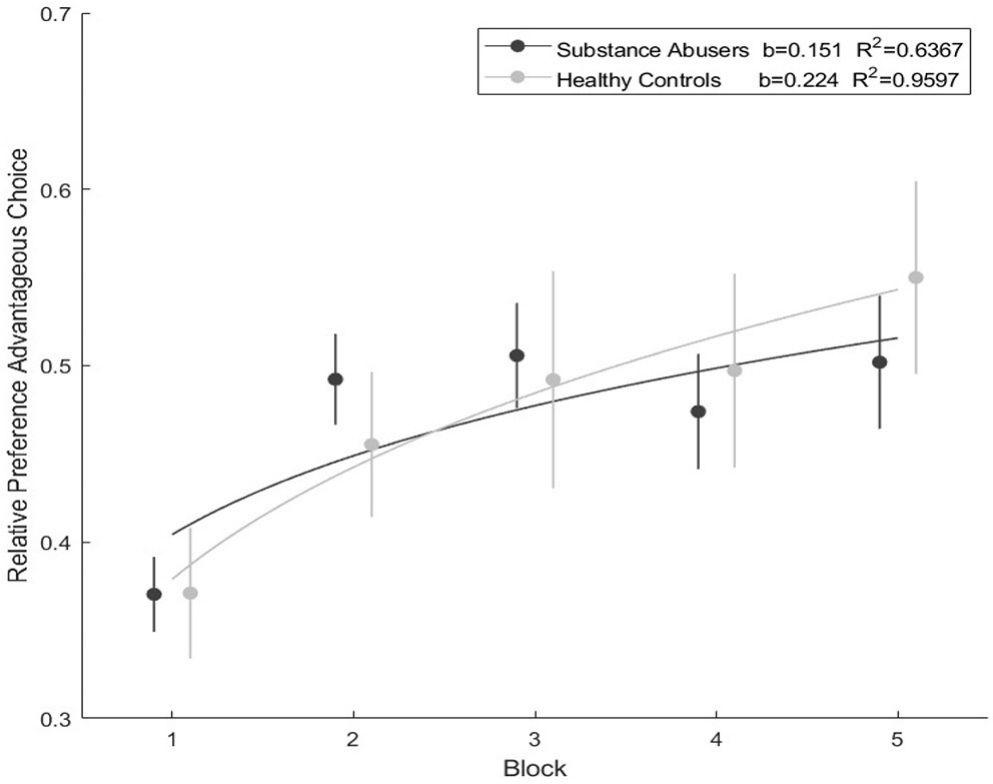
Iowa Gambling Task performance during the five blocks and the mathematical model fit.

When evaluating the results of the IGT, the healthy control group presented a greater preference for the advantage choice than the substance abuser group, but with non-significant differences. The mathematical model demonstrated a better fit for healthy controls (*b* = 0.227, *R*^2^ = 0.9398) than substance abusers (*b* = 0.151, *R*^2^ = 0.6367) (see Figure 3). The results of the repeated measures test found no differences in the performance of the five blocks (substance abusers: χ^2^ = 5.37, *gl* = 4, *p* = 0.251, η^2^ = 0.198; healthy Controls: χ^2^ = 6.69, *gl* = 4, *p* = 0.153; η^2^ = 0.267).

### Columbia Card Task Results

When evaluating the results of the CCT-hot task, the healthy controls chose a greater number of cards in comparison with substance abusers, however this tendency was not significant. We identified this pattern while evaluating the number of “loss” cards (*one card: Z* = −0.407, *p* = 0.684; three *cards: Z* = −0.455, *p* = 0.649); the number of points that could win on each card (*10 points: Z* = −0.487, *p* = 0.626; *30 points*: *Z* = −0.112, *p* = 0.911); and the number of points that could be lost if a “loss” card was chosen (*250 points*: *Z* = −0.056, *p* = 0.955; *750 points*: *Z* = −0.208, *p* = 0.836).

In the most favorable decision scenario (gain = 30; loss = −250; risk = 1), and in the most unfavorable decision scenario (gain = 10; loss = −750; risk = 3), substance abusers tended to select more cards than healthy users in both scenarios, though these differences were not significant (all *p*-values > 0.05).

**Figure 4:**
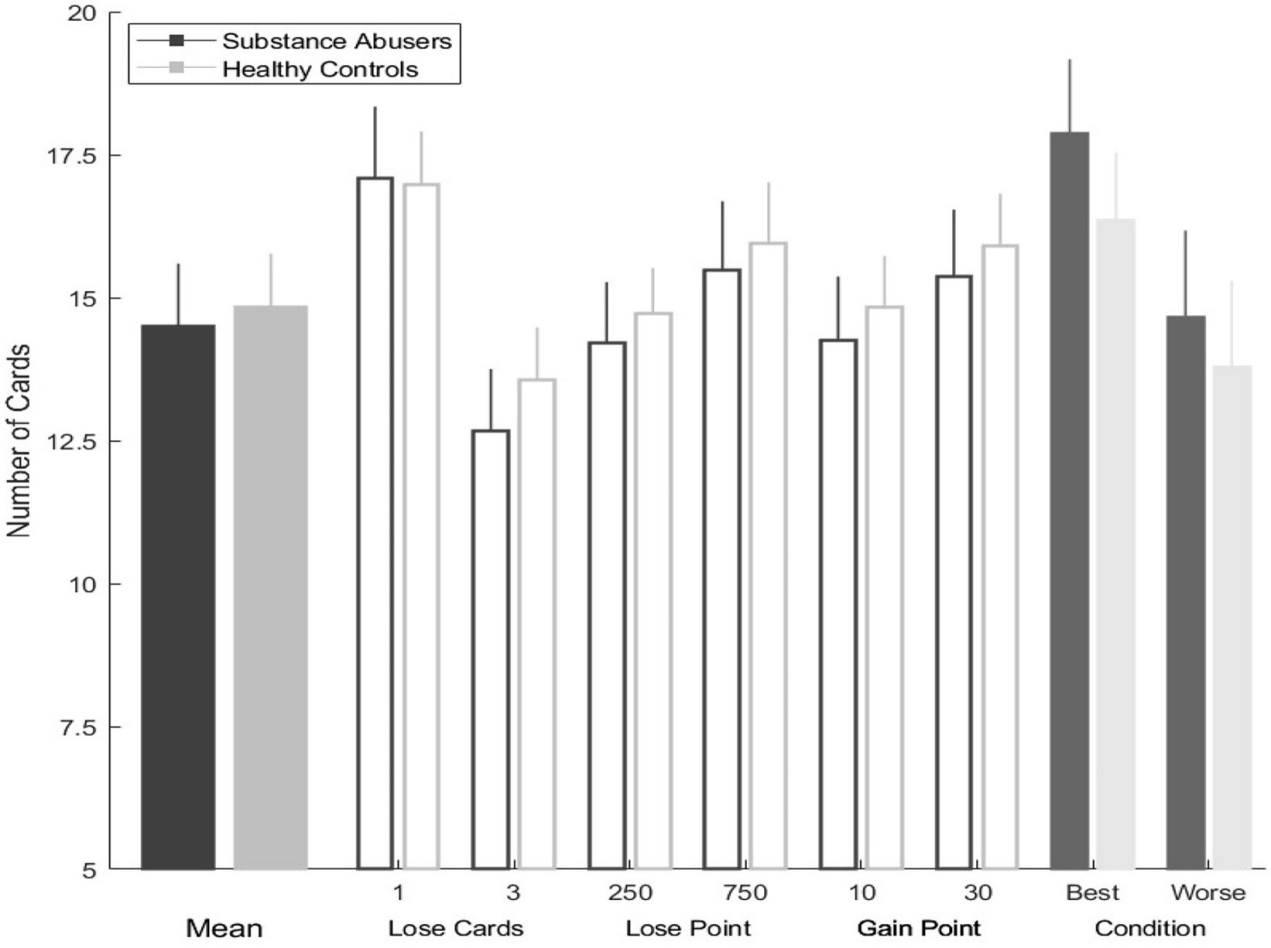
Columbia card task hot version performance in each condition by group.

The repeated measures test displayed statistically significant differences for the performance of the six manipulations: number of loss cards (one and three), gain points (10 and 30), loss points (250 and 750), along with lose points and gain points (substance abusers: χ^2^ = 47.2, *gl* = 5, *p* = 0.000, η^2^ = 1.74; healthy group: χ^2^ = 38.7, *gl* = 5, *p* = 0.000, η^2^ = 1.54) (see Figure 4).

The analysis of the CCT-cold task revealed that the substance abusers choose more cards than the healthy control group, but this tendency was not significant. We identified this pattern while evaluating the number of “loss” cards (*one card: Z* = −1.47, *p* = 0.140; three *cards: Z* = −1.07, *p* = 0.285); the number of points that could win on each card (*10 points: Z* = −1.48, *p* = 0.137; *30 points*: *Z* = −1.13, *p* = 0.257); and the number of points that could be lost if a “loss” card was chosen (*250 points*: *Z* = −1.22, *p* = 0.222; *750 points*: *Z* = −1.47, *p* = 0.140).

In the most favorable decision scenario (gain = 30; loss = −250; risk = 1), and in the most unfavorable decision scenario (gain = 10; loss = −750; risk = 3), substance abusers tended to select more cards than healthy users in both scenarios, but these differences were not significant (all *p*-values >0.05).

**Figure 5:**
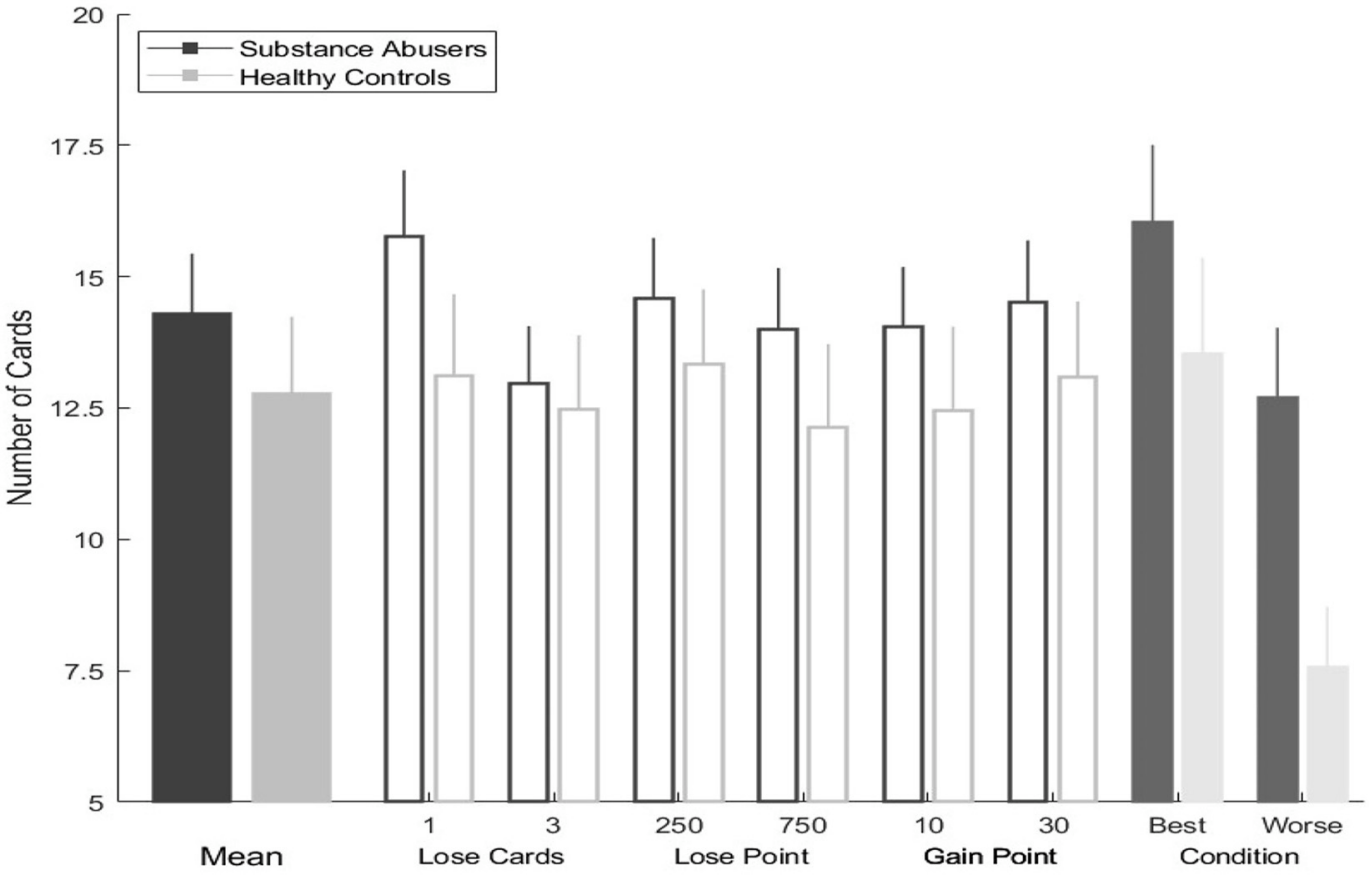
Columbia card task cold version performance in each condition by group.

The repeated measures test produced statistically significant differences in the performance of the manipulations of loss cards, lose points and points gained in substance abusers (χ^2^ = 21.8, *gl* = 5, *p* = 0.001, η^2^ = 0.808), but not in healthy Controls (χ^2^ = 8.06, *gl* = 5, *p* = 0.153, η^2^ = 0.322) (see Figure 5).

### Correlation and Factor Analysis Among the Behavioral Tasks

The matrix correlation showed statistically significant results between: BART-I and IGT-I (*r* = −0.269, *p* = 0.049); CCT-cold and *IGT-III* (*r* = −0.319, *p* = 0.019); CCT-cold and IGT-IV (*r* = −0.273, *p* = 0.046). Following the low *r* values displayed by the correlations, a Varimax rotation technique was used at all levels of the factor analysis. We found three factors with eigenvalues over 1.00 that accounted for 72.99% of the total variance. The Kaiser-Meyer-Olkin measure indicated that the analysis was the minimum acceptable value (*KMO* = 0.691), and Bartlett's test of sphericity revealed statistically significant results (χ^2^ = 246.74, *p* < 0.000). Factor 1 explained 32.92% of the variance, including the blocks III, IV, V of IGT, and the loaded weakly block II (factor loading = 0.618). Factor 2 accounted for an additional 18.47% of the variance, including all BART blocks. Factor 3 explained 11.78% of the variance, including IGT blocks I and II, and the loaded weakly DD (factor loading = −0.453). Factor 4 accounted for an additional 9.81% of the variance, including the CCT-hot and the CCT-cold (see Table 3).

**Table 3:** Exploratory factor analysis: Table for the decision-making tasks.

	**Rotated loadings**		
	**Factor 1**	**Factor 2**	**Factor 3**	**Factor 4**
IGT-I			0.804	
IGT-II	0.618		0.645	
IGT-III	0.896			
IGT-IV	0.879			
IGT-V	0.800			
BART-I		0.915		
BART-II		0.888		
BART-III		0.608		
DD			−0.453	
CCT-hot				0.836
CCT-cold				0.688
Eigenvalue	3.622	2.032	1.296	1.080
% Total variance	32.92%	18.47%	11.78%	9.81%

*IGT, Iowa Gambling Task, average of advantageous selection by 20-trials blocks; BART, Balloon Analog Risk Task, average number of adjusted balloons; CCT-cold and CCT-hot, Columbia Card Task, average number of card selections; DD, AUC of delay discounting*.

### CART Algorithm Results

The decision tree exhibited that the best factors for classifying the participants' performances were IGT block I, IGT block V, and CCT-cold task. The results calculated revealed in the first decision rule: scores ≥0.525 in the IGT Block 1 are related to healthy controls (*GI* = 0.062), and the scores <0.525 for the IGT Block 1, indicated that the participants belong to the substance abuse group (*GI* = 0.175). After that decision, in the second rule: scores greater or equal to 2.93 in the CCT-cold suggest that these participants belong to the healthy control group (*GI* = 0.143), and the scores lesser than 2.93 for the CCT-cold, that the participants belong to substance abuse group (*GI* = 0.000).

**Figure 6:**
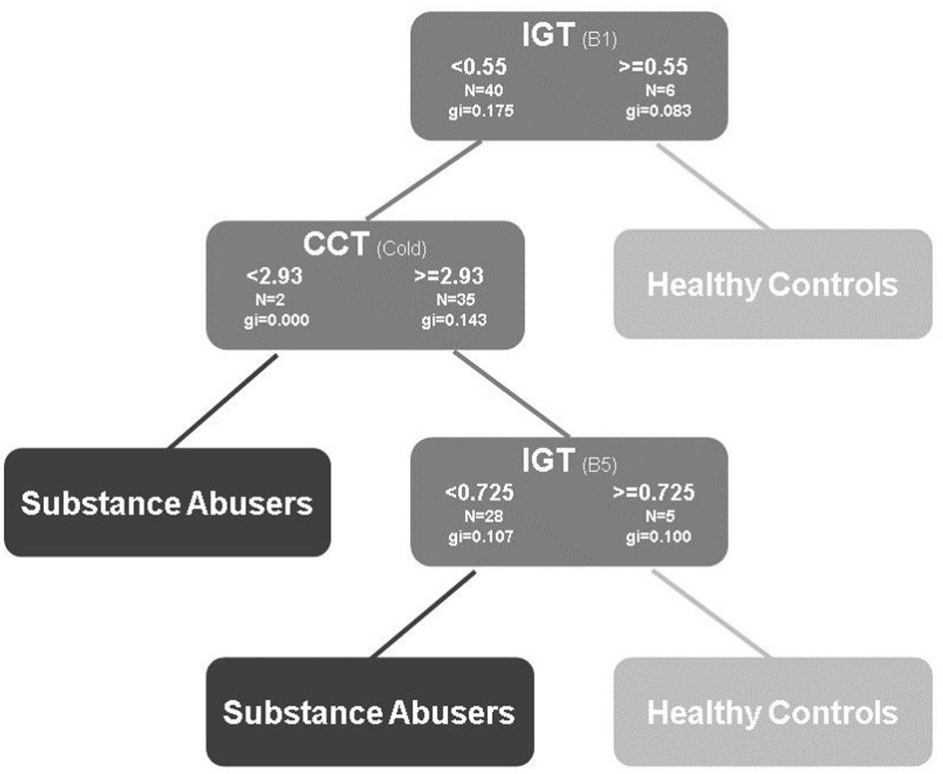
Decision tree using: IGT, Iowa Gambling Task, advantageous selections by 20-trials blocks; BART, Balloon Analog Risk Task, average number of adjusted balloons; CCT-cold and CCT-hot, Columbia Card Task, average number of card selections; DD, AUC of delay discounting.

Finally, after the second decision, the third rule: scores ≥0.725 in the IGT Block V suggest that the participants belong to the healthy control group (*GI* = 0.100), and the scores <0.725 for the IGT Block V that the participants belong to substance abuse group (*GI* = 0.107) (see Figure 6). The lack of accuracy of the classification tree was high (*L* = 0.24). This was attributed to sample size and the lack of differences between groups.

## Discussion

This study aimed to identify differences between substance abusers and healthy control participants in two uncertainty decision-making tasks (BART, IGT trial 1–40), three risk-taking tasks (CCT-hot, CCT-cold, IGT trial 41–100), and one impulsivity task (DD). The data analysis did not reveal any statistically significant differences, and small effect sizes among groups from the decision-making tasks. In consideration of the evidence demonstrated by the literature on each type of task (2, 6, 8, 15), these results were not expected; however, we are aware that we had a small sample size with insufficient power to detect such a difference (see Table 2).

The main challenge of an online study is to ensure that participants are paying sufficient attention to effectively complete surveys or cognitive tasks. For example, although we confirmed that healthy control participants finished all the tests, we could not guarantee whether they understood the goal of each task. Similarly, we could not determine whether participants might have had doubts during their completion. Regarding the performance difference between substance abuse participants, such divergence might have resulted from the varying abstinence time displayed by the distribution dispersion. Therefore, we suggest that future studies control these variables and increase the sample size by contrasting poly-drug abuse, mono-drug abuse, and within-task effects, into their own separate groups (healthy controls and substance abusers). For example, some researchers studying discounting tasks have found no differences between mono-drug abuse and poly-drug abuse (35, 36).

These results reveal statistically significant differences between the groups related to drug use, tobacco, marijuana (cigarette), and crystal meth consumption as the principal impact drug. However, the effects of crystal meth are still not clear enough. It has recently been found that the acute effects of methamphetamines improved attention and inhibition during specific cognitive procedures. Nevertheless, a minority of cognitive measures, such as visuospatial perception, attention, inhibition, working memory, and long-term memory has only observed the long-term effects of methamphetamines (37). In our study, the analysis did not show significant differences between groups in the delay-discounting task, but we found better fitting and lesser *k* values in healthy controls when compared to substance abusers. While assessing the IGT task, the learning model revealed a better fit for healthy control participants than substance abusers, indicating an improved learning process. This finding acknowledges that healthy control participants learned how to choose advantageous cards. We provided new information about methamphetamine use.

Nevertheless, we recommend in future studies to randomize the task order, in this study, we used a web platform where we could not randomize the tasks, and this factor can in turn influence the decision-making, specially the IGT transitions from under ambiguity to under risk. For example, Shapiro et al. (38) studied the carryover effect of gains and losses in a previous task to a risk-taking decision task, and an impulsivity task. In this study, experienced losses raised risk-taking decisions but did not affect impulsivity choices in delay discounting tasks. The order that we used could affect all the risk-taking tasks (IGT 40-100, CCT-hot, and CCT-cold).

The number of cards selected in the CCT-hot and the CCT-cold tasks fluctuated as a function of losing cards; the losing points and winning points indicated that both groups paid attention to these changes. We collected the average of response latency (sec) in each trial by task. We found significant differences between the groups in the CCT-cold, CCT-hot, and IGT. This is important due the fact we were with the clinical population during all the tasks, and we resolved all the doubts. We conclude that the healthy controls paid attention to the tasks, and they had diverse performances according to the task, see Supplementary Material.

According to the balloon probabilities, the results from the BART task displayed differences in participant performance across both groups. This descriptive pattern follows a similar tendency reported by other clinical studies (8). As in previous studies regarding alcohol use disorder (39–41) these results should be considered while implementing substance abuse treatment programs. For example, creating cognitive bias modification tasks that can alter the decision-making process among meth-abusers. In the clinical field, it is common for self-reported tests to be used most frequently. However, incorporating more cognitive tasks could increase the effectiveness of treatments and function as a more objective evaluation of substance-related problems.

The second objective of the present research was to evaluate the correlation between the behavioral test battery and to identify predictive factors of substance abuse. While estimating the correlation values among the cognitive tasks, the results demonstrated scarcely correlated procedures and a small effect size. Among the uncertainty tasks (i.e., BART, IGT Block I, and IGT Block II), we had expected strong correlations, however, the analysis displayed significant correlations with low values among these variables. These results were similar to those reported by Xu et al. (17), but contradicted the results reported by Buelow and Barnhart (22). In addition, we found that IGT blocks III and IV correlated with CCT-cold. This outcome is related to the risk-taking process and is comparable to the study by Brunell and Buelow (2015). Further investigation is needed to study the contrast between performance in IGT and its relation to other tasks. Such inquiry is also needed to clarify the learning process in uncertain environments as well as one's transition to riskier environments.

We had assumed that impulsivity would be more related to the risk-taking process than the uncertainty decision-making process. This assumption was made because the elements of the decision process were established by the delay and amount in discounting tasks. Surprisingly, the results showed a relationship between the delay discounting procedure and the IGT blocks I and II, similar to the effects reported by Xu et al. (17). Although this study used the net score in the IGT task.

In the factor analysis, we found four factors. First, each decision task evaluated a different decision-making process, even delay discounting loaded weakly to the third factor; when we performed oblimin rotation to verify our factors, we found the same, but the factor loadings decreased (see Supplementary Material). Second, this rotation of factors was comparable to the study by Buelow and Blaine (20). Indeed, uncertainty and risk behaviors may recruit different brain systems or recruit a common brain mechanism at different levels (18). Additionally, the decision-making process under risky scenarios depends more on executive functioning than on uncertainty processes (42). According to these opposing views about the decision-making process, we encourage future researchers to explore executive tasks (working memory, inhibition, and shifting) with decision-making tasks. Furthermore, we recommend increasing the sample size according to MacCallum et al. (34). Considering the low communality and the number of variables, we recommend a sample size of 200 for a 95% convergent and admissible solution.

The CART algorithm helped identify that the accurate predictors of substance abuse were the IGT (blocks I, and V) and CCT-cold tasks. Although we did not find any differences among the groups, and considering the small sample size, the findings allowed us to understand the complexity of the decision-making process. For example, the presented results lay the foundation for exploring the relationship between CCT and IGT tasks as favorable predictors of risky behavior. According to the logistic regression we performed for contrasting the CART algorithm, none of the behavioral tasks were significant predictors, with a global percentage of classification 64.8%; however, the performance in all the blocks of IGT had the highest B values, thus supporting the algorithm (see Supplementary Material). We consider that this type of analysis can be valuable because the information from the test sequence can be used in the future to evaluate the participants in steps or stages using several decision-making tasks such as IGT first and after CCT-cold task. We recommend a sensitivity and specificity analysis with receiver operating characteristic (ROC) curves with these tasks. This analysis can provide an accurate classification.

We suggest evaluating the magnitude effect with delay discounting tasks by analyzing the probabilistic outcomes and losses. In the case of the Columbia card task, it might be interesting to study the affective system (CCT-hot) and its relationship with social discounting as a measure of attachment to others. Even with risky and uncertain tasks, introducing effort discounting may be interesting. For instance, comparing several cognitive tasks such as BART or IGT, and not just the discounting procedures (43). As our results suggest, crystal meth and methamphetamines are drugs that affect several cognitive tasks. For this reason, when evaluating substance abusers, we recommend considering the comorbidity with other mental disorders, contrasting several impact drugs, time of abstinence, and using scales to assess psychiatric, neurological, or medical conditions.

Considering our interest in working with substance abusers, we expected significant differences in the demographic variables such as age, education, and income. Nevertheless, for future studies, we recommend increasing the sample size, evaluating the female population, and using Propensity Score Matching to match the substance abusers and healthy controls with higher precision. Education level is another essential variable to consider because substance abuse can influence school attendance and academic achievement (44, 45). In the current study, the substance abusers had lower levels of education. We therefore recommend considering education level as a covariate. Nevertheless, we did not find any significant correlations between the cognitive tasks and level of education, (see correlation matrix in the Supplementary Material).

Finally, we conclude that many factors affect the complex decision-making process in the clinical and healthy population. Such factors include uncertainty tasks (BART and IGT in the 1–40 blocks); risk cues in the tasks (number of losses, number of gains, and cards of loss in CCT tasks), and time of delivery (DD task).

## Data Availability Statement

The raw data supporting the conclusions of this article will be made available by the authors, without undue reservation.

## Ethics Statement

The studies involving human participants were reviewed and approved by the Sonora Institute of Technology PROFAPI Review Board (ID 84). The patients/participants provided their written informed consent to participate in this study.

## Author Contributions

DM and LA-C designed the study and wrote the protocol. LA-C conducted literature searches and provided summaries of previous research studies. DM wrote the first draft of the manuscript. AT-F conducted the statistical analysis. All authors contributed to and have approved the final manuscript.

## Funding

This study received financial support from the Sonora Institute of Technology PROFAPI_2021_0017.

## Conflict of Interest

The authors declare that the research was conducted in the absence of any commercial or financial relationships that could be construed as a potential conflict of interest.

## Publisher's Note

All claims expressed in this article are solely those of the authors and do not necessarily represent those of their affiliated organizations, or those of the publisher, the editors and the reviewers. Any product that may be evaluated in this article, or claim that may be made by its manufacturer, is not guaranteed or endorsed by the publisher.
