# Supplementary material for: Decision-Making Under Risk and Uncertainty by Substance Abusers and Healthy Controls
Source: Front Psychiatry. 2022 Jan 28;12:788280. doi: 10.3389/fpsyt.2021.788280 (PMC8833085; doi:10.3389/fpsyt.2021.788280)
Supplement: Supplementary file 1 [file Data_Sheet_1.pdf]

Supplemental material

Correlation Matrix among the tasks, age, years of education, and time of abstinence

	1	2	3	4	5	6	7	8	9	10	11	12	13	14
1.BART-I	1		.											
2.BART-II	.377**	1												
3.BART-III	.380**	.802**	1											
4.CCT-cold	.111	-.022	-.091	1										
5.CCT-hot	.047	-.118	-.075	.279*	1									
6.DD	-.147	-.171	-.249	-.103	-.100	1								
7.IGT-I	-.269*	-.209	-.198	-.083	-.113	.059	1							
8.IGT-II	-.180	-.015	.036	-.185	-.029	.034	.649**	1						
9.IGT-III	-.200	-.150	-.071	-.319*	.061	.128	.561**	.692**	1					
10.IGT-IV	-.229	-.117	-.053	-.273*	-.082	.179	.322*	.561**	.713**	1				
11.IGT-V	-.209*	-.144	-.164	-.145	.123	.187	.222	.501**	.629**	.777**	1			
12.Years of education	.205	-.050	-.076	-.014	.024	-.117	.023	.079	-.026	.051	.046	1		
13. Age	.200	-.066	-.044	-.004	-.143	.076	.224	.090	.155	.079	.065	-	1	
14.Time of abstinence	.041	-.050	.162	.207	.159	.045	.090	.132	.155	.111	-.000	-	-	1

$^{*}p \leq .05$; $^{**}p \leq .01$;

Exploratory Factor Analysis: Oblique Rotations of the DATA

	Rotated loadings			
	1	2	3	4
BART- I		.635		
BART-II		.884		
BART-III		.902		
CCT-cold			.705	
CCT-hot			.822	
DD				.516
IGT-I	.442			-.755
IGT-II	.724			-.608
IGT-III	.868			
IGT-IV	.896			
IGT-V	.874			
Eigenvalue	3.622	2.032	1.296	1.080
% Total variance	32.93%	18.47%	11.78%	9.812%

Note. IGT- Iowa Gambling Task, average of advantageous selection by 20-card blocks of trials; BART- Balloon Analog Risk Task, average number of adjusted balloons; CCT-cold and CCT-hot Columbia Card Task, average number of card selections; DD-AUC of delay discounting.

Binary Logistic Regression Analyses: Predicting substance abusers and healthy controls

	B	*SE*	*Wald*	*sig*	*Exp (B)*	*95% CI*
Model Nagelkerke *R = 195*						
BART- I	-.088	.289	092	.762	.916	.520 - 1.615
BART-II	.166	.159	1.093	.296	1.181	.865 - 1.612
BART-III	-.078	.106	.541	.462	.925	.752 - 1.138
CCT-cold	-.074	.046	2.588	.108	.929	.848 - 1.016
CCT-hot	.023	.054	.176	.675	1.023	.920 - 1.138
DD	-2.224	1.280	3.016	.082	.108	.009 - 1.331
IGT-I	1.229	2.485	.244	.621	3.416	.026 - 445.726
IGT-II	-2.456	2.239	1.203	.273	.086	.001 - 6.910
IGT-III	-1.864	2.133	.764	.382	.155	.002 - 10.135
IGT-IV	1.362	2.054	.440	.507	3.904	.070 - 218.879
IGT-V	.911	1.919	.225	.635	2.486	.058 - 106.902

Response latency (sec) in each trial by task

	BART		CCT-cold		CCT-hot		DD		IGT	
	SA	HC	SA	HC	SA	HC	SA	HC	SA	HC
M	2.788	2.116	8.725	5.335	8.986	4.984	5.605	5.863	2.871	0.848
SD	1.422	0.891	4.880	4.939	6.080	2.203	2.109	4.004	2.623	0.392
Z	454.5		531.5		577.0		413.0		663.0	
p	0.119		0.004		< .001		0.401		< .001	
